# Optimizing engineered TCR T cell therapy for synovial sarcoma

**DOI:** 10.1186/2051-1426-3-S2-P159

**Published:** 2015-11-04

**Authors:** Sandra D'Angelo, Melinda Merchant, Luca Melchiori, Hua Zhang, Lini Pandite, Tom Holdich, Gwendolyn Binder-Scholl, William Tap, Marylene Fortin, Yoav Peretz, Matthew Wright, Paul Meyers, Rafael Amado, Bent Jakobsen, Crystal Mackall

**Affiliations:** 1 Memorial Sloan Kettering Cancer Center, New York, NY, USA; 2 National Cancer Institute, Bethesda, MD, USA; 3 Adaptimmune, Abingdon,, United Kingdom; 4 Adaptimmune, Philadelphia, PA, USA; 5 Immunecarta Services, Montreal, USA

## 

Relapsed or metastatic synovial sarcoma remains a significant unmet medical need. NY-ESO-1 is an attractive target for sarcoma, since it is expressed in approximately 70% of synovial sarcomas but not on vital tissues. We generated NY-ESO^c259^, a human-derived affinity-enhanced T-cell receptor (TCR) that recognizes the NY-ESO-1– derived SLLMWITQC peptide in complex with HLA-A*02. Adoptive transfer of autologous T cells expressing NY-ESO^c259^ was clinically tested in patients with metastatic synovial cell sarcoma and melanoma whose tumor expressed NY-ESO-1 protein at an intensity of ≥2+ and in 50% of cells by immunohistochemistry; infusion followed preconditioning with fludarabine and cyclophosphamide, and systemic IL-2 support was given[1]. We subsequently initiated a study in relapsed/metastatic synovial sarcoma, with an updated T cell manufacturing method utilizing anti-CD3/28 paramagnetic microbeads for simultaneous activation and co-stimulation of T cells, and genetic modification with a lentivector (NCT01343043). No IL-2 support was given. Outcomes of this study were recently presented[2]; clinical data demonstrate safety, objective clinical responses in 50% of patients, and improved and durable engineered T cell persistence. This study has been extended to include two additional cohorts to evaluate the removal of fludarabine on tumor responses (Cohort 3) and to evaluate responses in patients who have antigen positive tumor below the current ≥2+ and 50% threshold (Cohort 2).

To better understand the lineage and functional characteristics of the persisting engineered T cells, as well as to understand the role of the starting material and manufacturing method in modifying the T cell phenotype and its fate once *in vivo*, we performed multiparameter flow analysis. Baseline and post infusion PBMCs, as well as manufactured product, were analyzed to evaluate memory and exhaustion markers (e.g. CD45RA, CCR7, PD-1), polyfunctionality/cytotoxicity markers (e.g. IFN-γ and Granzyme B), and costimulatory markers (e.g. OX40, ICOS, CD28). TCR expression was measured by pentamer. Hierarchical cluster analysis was applied to identify trends of expression of surface markers and their correlation with final cell product profile, persistence and clinical response. An update of Cohort 1 safety, efficacy and correlative biomarker analyses, and a status update on Cohorts 2 and 3 will be presented.

## Trial registration

ClinicalTrials.gov identifier NCT01343043.
